# Whole‐Brain Monosynaptic Inputs to and Axonal Projections From Posterior Insular Cortex GABAergic Neurons in Mice

**DOI:** 10.1002/cns.71102

**Published:** 2026-09-04

**Authors:** Wan‐Qiao Qi, Xin‐Yue Zhang, Wei‐Xiang Ma, Wei‐Min Qu, Jia‐Qi Shi, Zhi‐Li Huang, Yu‐Lan Zhu

**Affiliations:** ^1^ Department of Neurology The Second Affiliated Hospital of Harbin Medical University Harbin China; ^2^ Department of Anesthesiology, Zhongshan Hospital Fudan University Shanghai China; ^3^ State Key Laboratory of Brain Function and Brain Disorders and MOE Frontiers Centre for Brain Science, Department of Pharmacology, School of Basic Medical Sciences, Institutes of Brain Science, Joint International Research Laboratory of Sleep, Shanghai Medical College Fudan University Shanghai China

**Keywords:** anterograde tracing, GABAergic neurons, posterior insular cortex, retrograde tracing

## Abstract

**Background:**

The posterior insular cortex (PIC) is a multimodal sensory integration center, and the GABAergic neurons are associated with functions such as pain perception, emotional regulation, and fear memories encoding. However, the whole‐brain connectivity to PIC GABAergic neurons remains unclear.

**Objective:**

To map the whole‐brain input–output connectivity of PIC GABAergic neurons.

**Methods:**

This study combined cell‐type‐specific retrograde/anterograde viral tracing systems with Cre/loxP genetic technology to perform a comparative analysis of the localization and quantification of monosynaptic inputs and axonal projections of PIC GABAergic neurons.

**Results:**

Retrograde tracing identified 45 input nuclei, with densest inputs from the piriform cortex (Pir) and secondary somatosensory cortex (S2) of the isocortex, thalamic nuclei including the posterior thalamic nuclear group (PO) and parafascicular thalamic (PAF), exhibiting ipsilateral preference. Anterograde tracing revealed 18 output targets, with densest projections to the caudate putamen (CPu), external globus pallidus (GPe), and ventral pallidum (VP) of the basal ganglia and multiple subnuclei of the amygdala.

**Conclusions:**

PIC GABAergic neurons receive dense inputs from the isocortex and thalamus and send prominent projections to the basal ganglia and amygdala, with these brain regions constituting key hubs of the whole‐brain connectivity network. This connectivity pattern provides a novel anatomical perspective for elucidating their roles in physiological behavior regulation.

## Graphical abstract

We mapped whole‐brain inputs and outputs of PIC GABAergic neurons. This study revealed that PIC GABAergic neurons receive dense inputs from the isocortex and thalamus and send strong projections to the basal ganglia and amygdala, with these regions constituting key hubs of the connectivity network. This connectivity pattern provides a novel anatomical perspective for elucidating the roles of PIC GABAergic neurons in physiological behavior regulation.

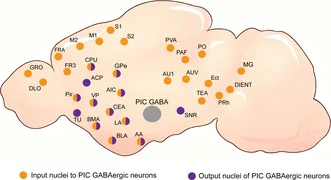

## 1 Introduction

The posterior insular cortex (PIC) is a critical substructure of the insular cortex, located posterior to the central insular sulcus. Its functions have evolved from a simple sensory relay station to a complex center for cognitive and behavioral regulation. The PIC consists of glutamatergic and GABAergic neurons. Glutamatergic neurons serve as the main long‐range projection neurons of the PIC, involved in encoding interoceptive information, taste perception, pain processing, and proprioception [1, 2, 3, 4]. Traditionally, GABAergic neurons were thought to regulate local microcircuits, but recent studies show they have diverse functions, including modulating neuropathic pain [5], stress‐induced tachycardia [6] and fear memory consolidation [7]. Some GABAergic neurons even possess long‐range projection capabilities [8, 9, 10, 11]. However, the complete whole‐brain input and output connectivity of PIC GABAergic neurons remains unclear. Therefore, delineating their upstream and downstream projection patterns is crucial for understanding their roles in various physiological functions.

In this study, we utilized modified rabies virus (RV) and recombinant adeno‐associated virus (rAAV) vectors combined with Cre/loxP genetic strategies to overcome the traditional tracing limitations. We identified 45 input and 18 output nuclei, revealing asymmetric connectivity with the isocortex, amygdala, basal ganglia, and thalamus. Our viral tracing data further revealed that PIC GABAergic neurons predominantly receive upstream inputs from the isocortex and thalamus, while their downstream axons mainly project to the basal ganglia and amygdala. This distinct connection pattern constitutes a critical anatomical foundation for deciphering the circuit‐level integration mechanisms of PIC GABAergic neurons across diverse physiological functions. This study further systematically summarizes the potential functional regulations in which these associated neural circuits may be involved, including physiological activities such as sleep–wake regulation, pain and thermal perception, and conditioned fear memories. It provides a structural framework for elucidating the neural pathways related to PIC GABAergic neurons and offers new insights for the development of novel drugs and the treatment of related clinical disorders.

## 2 Materials and Methods

### 2.1 Animals

Adult GAD2‐IRES‐Cre mice (C57BL/6J background, 8–10 weeks, 25–27 g, Jackson Lab Stock no. 010802) that specifically target almost all brain GABAergic neurons [12] and wildtype (WT) littermates (same age/weight) were used. Animals were housed under standard conditions (12‐h light/dark cycle, 25°C ± 0.5°C, food and water ad libitum).

### 2.2 Viruses

BrainVTA (Wuhan, China) packaged the viral vectors. The titers of AAV‐EF1α‐DIO‐TVA‐EGFP, AAV‐EF1α‐DIO‐RvG, and RV‐EnvA‐ΔRG‐DsRed (EnvA‐pseudotyped, RG‐deleted, DsRedexpressing) were 5 × 10^12^, 5 × 10^12^, and 2 × 10^8^ genomic copies/mL, respectively. All three AAVs were packaged in serotype 2/9. The AAV2/9‐EF1α‐DIO‐ChR2EYFP titer was ~3 × 10^12^ copies/mL.

### 2.3 Viral Injections

Mice were anesthetized with 1.5%–2% isoflurane and placed in a stereotaxic frame. For retrograde tracing, a helper virus cocktail was prepared by mixing AAV‐EF1α‐DIO‐TVA‐EGFP and AAV‐EF1α‐DIO‐RvG at a 1:1 ratio (v/v), and 150 nL of this mixture (approximately 75 nL of each virus) was injected into the PIC (AP: −0.4 mm; ML: +4.1 mm; DV: −4.25 mm) at 40 nL/min in GAD2‐IRES‐Cre mice or WT. Three weeks later, 300 nL of RV‐EnvA‐ΔRG‐DsRed was injected into the same site [13, 14, 15, 16]. Perfusion was performed 1 week after RV injection [17]. For anterograde tracing, 100 nL of rAAV‐EF1α‐DIO‐ChR2‐EYFP [18, 19, 20] was injected into the PIC in GAD2‐IRES‐Cre mice, and mice were perfused 3 weeks later [21]. Inclusion criteria for the 12 GAD2‐IRES‐Cre and 4 wildtype mice that received injections were: injection centered in PIC (Allen atlas), robust eYFP expression in PIC GABAergic neurons (rAAV) or adequate starter cells (RV), and no leakage into adjacent regions. Exclusion criteria: off‐target injection, weak/absent expression, poor perfusion/tissue damage, or nonspecific labeling. Ultimately, 8 GAD2‐IRES‐Cre mice met criteria (input *n* = 4, output *n* = 4). Four wildtype mice injected with the same viruses served as negative controls (no Cre‐dependent expression).

### 2.4 Histology and Immunostaining

Mice were transcardially perfused with PBS and 4% paraformaldehyde. Brains were postfixed, cryoprotected in 30% sucrose, and sectioned at 30 μm (CM1950, Leica, Germany). Sections were washed in PBS, blocked with immunostaining blocking buffer for 60 min at RT, then incubated overnight at 4°C with mouse anti‐GABA primary antibody (1:500, Sigma‐Aldrich, SAB4200721) in PBS with 2.5% Triton X‐100 (T9284; Sigma‐Aldrich). After washing, sections were incubated with secondary antibody (Alexa Fluor 647 goat anti‐mouse IgG, 1:1000, Abcam) for 2 h at RT, washed 3× 10 min in PBS, stained with DAPI (1:10000, Sigma‐Aldrich D9542) for 10 min at RT, washed, mounted, and coverslipped.

### 2.5 Data Analysis

For whole‐brain tracing, images were acquired with 10× or 20× objective on a slide scanner (VS200, Olympus). To map starter neurons in PIC, each section was registered to the Allen Mouse Brain Atlas using ImageJ. Coronal sections were matched to atlas plates by anatomical landmarks (hippocampus, corpus callosum) to determine rostrocaudal level; then, semi‐manual rigid alignment was done with BigWarp/TurboReg using 3–5 landmarks (midline, fiber tracts, ventricular corners). After alignment, we counted total starter cells and mapped their distribution.

For input nuclei quantification, dsRed‐labeled somata in each nucleus were automatically quantified in ImageJ, manually verified, and expressed as a percentage of total whole‐brain dsRed^+^ cells.

For axonal projections, we applied criteria [22, 23]: the axonal structures were defined as varicosities when their transverse diameter greater than 0.5 μm. Axons bearing such varicosities were considered as projecting to downstream brain regions, as opposed to passing axonal fibers lacking such structures. Varicosities located on straight, smooth axonal segments without branching or local arborization (i.e., typical fibers of passage) were excluded from quantification; only varicosities on axon collaterals or within terminal fields were counted. Semi‐automated quantification of EYFP varicosities used ImageJ (1.53c): images were 8‐bit, Gaussian‐blurred (sigma = 1.0), size‐filtered for Feret's diameter > 0.5 μm, then maxima identified (noise tolerance = 15, edge maxima excluded), prominence threshold set, and manual correction removed false positives. Projection contribution was percentage of EYFP varicosities in each nucleus relative to whole‐brain total [24, 25]. Data are mean ± SEM.

## 3 Results

### 3.1 Identification of Monosynaptic Inputs to PIC GABAergic Neurons

**FIGURE 1:**
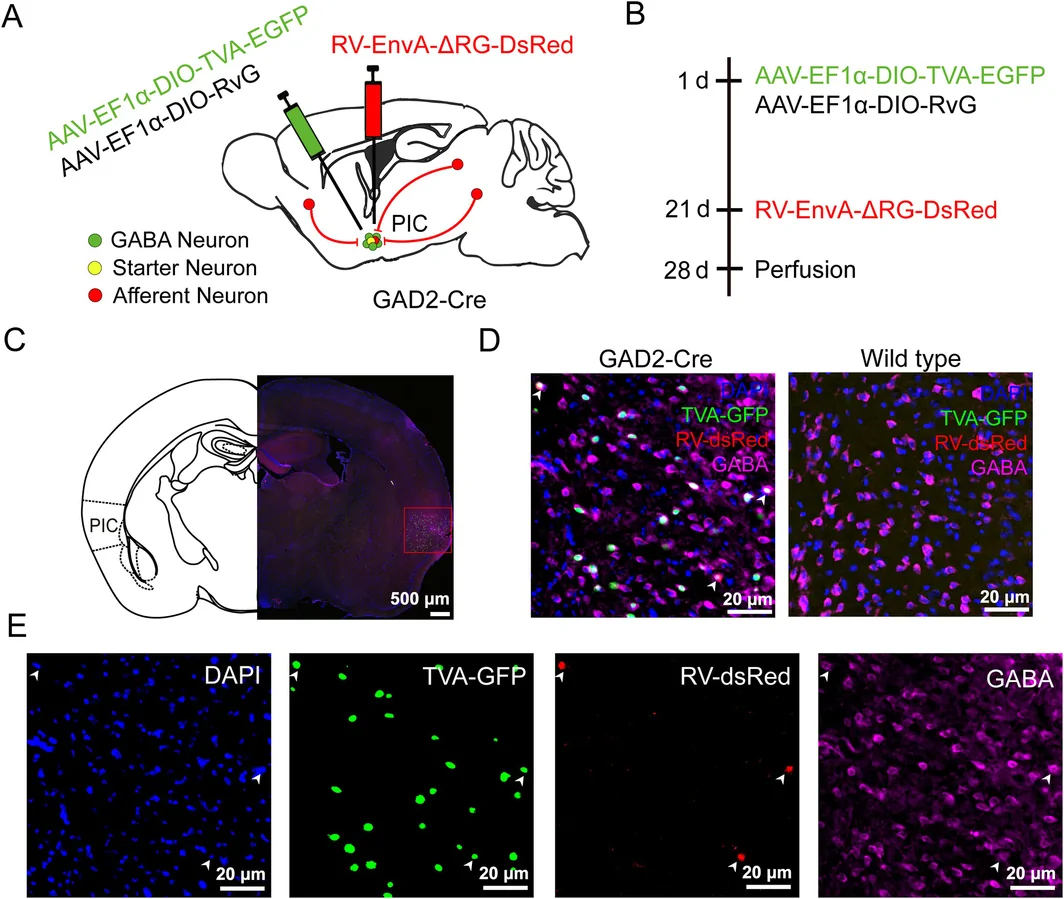
Monosynaptic input mapping to PIC GABAergic neurons and starter neuron visualization via rabies virus‐based tracing in GAD2‐IRES‐Cre mice. (A) Experimental schematic depicting helper virus injection (AAV‐EF1α‐DIO‐TVA‐EGFP and AAV‐EF1α‐DIO‐RvG) into the PIC of GAD2‐IRES‐Cre mice, followed by retrograde tracing with RV‐EnvA‐ΔRG‐DsRed. (B) Experimental timeline illustrating retrograde tracing of projections to the PIC. (C) Representative images showing the unilateral injection target GFP and DsRed expressing neurons in the PIC (red box) after helper virus and rabies virus injections in GAD2‐IRES‐Cre mice. (D) An enlarged view of the red boxed region in (C) shows that starter cells (marked by arrowheads) co‐expressed DAPI, GFP, DsRed and GABA immunoreactive signals in a GAD2‐IRES‐Cre mouse (left) and the absence of labeled cells in a wild‐type control (right). (E) Immunofluorescence image depicting individual channels of the merged image in left panel of (D) and showing expression of DAPI (blue), GFP (green), dsRed (red), GABA (pink) signals and starter neurons (marked by white arrows) in the PIC of GAD2‐IRES‐Cre mice.

To identify the whole‐brain monosynaptic inputs, we employed a rabies virus‐mediated retrograde trans‐synaptic tracing technique in GAD2‐IRES‐Cre mice that specifically express Cre recombinase. This viral tracing system has been validated to precisely and specifically label monosynaptic afferent signals received directly by starter neurons [21, 26]. Initially, we performed unilateral stereotaxic injection of two helper adeno‐associated viruses (AAV‐EF1α‐DIO‐TVA‐EGFP and AAV‐EF1α‐DIO‐RvG) into the PIC in GAD2‐IRES‐Cre mice or C57BL/6J mice. Three weeks later, RV‐EnvA‐ΔRG‐DsRed was injected into the same site. This modified rabies virus selectively infects TVA‐expressing cells and, with the assistance of RG, undergoes retrograde trans‐synaptic spread to presynaptic neurons. This enabled the co‐expression of enhanced green fluorescent protein (EGFP), the avian virus receptor TVA, and the rabies virus glycoprotein (RG) specifically in GABAergic neurons in the PIC (Figure 1A) [17]. One week postinjection, mice were transcardially perfused, and brain tissue samples were collected (Figure 1B).

Starter neurons were identified based on co‐localization of RV‐EnvA‐ΔRG‐DsRed and AAV‐EF1α‐DIO‐TVA‐EGFP signals (Figure 1C–E), while neurons positive only for DsRed were identified as presynaptic input neurons. To verify the neurochemical identity of the starter cells, immunofluorescence staining with an anti‐GABA antibody was performed in GAD2‐IRES‐Cre mice, confirming the specificity of the viral labeling strategy for GABAergic neurons (Figure 1D). All tissue sections were imaged and registered to the Allen Mouse Brain Atlas following fluorescence imaging. We mapped the location of starter cells and found that they were predominantly distributed in the middle PIC region, with the highest density observed between bregma −0.23 and −0.71 mm (Figure S1). The average number of starter cells per mouse was 39.36 ± 1.66 (Figure S2A). In WT mice subjected to the same injection strategy, no GFP or DsRed‐positive signals were observed, confirming that viral infection and the expression of EGFP and DsRed were strictly Cre‐dependent (Figure 1D). In summary, we successfully established the input network map of monosynaptic afferents targeting PIC GABAergic neurons.

### 3.2 Input Patterns of GABAergic Neurons in the PIC

**FIGURE 2:**
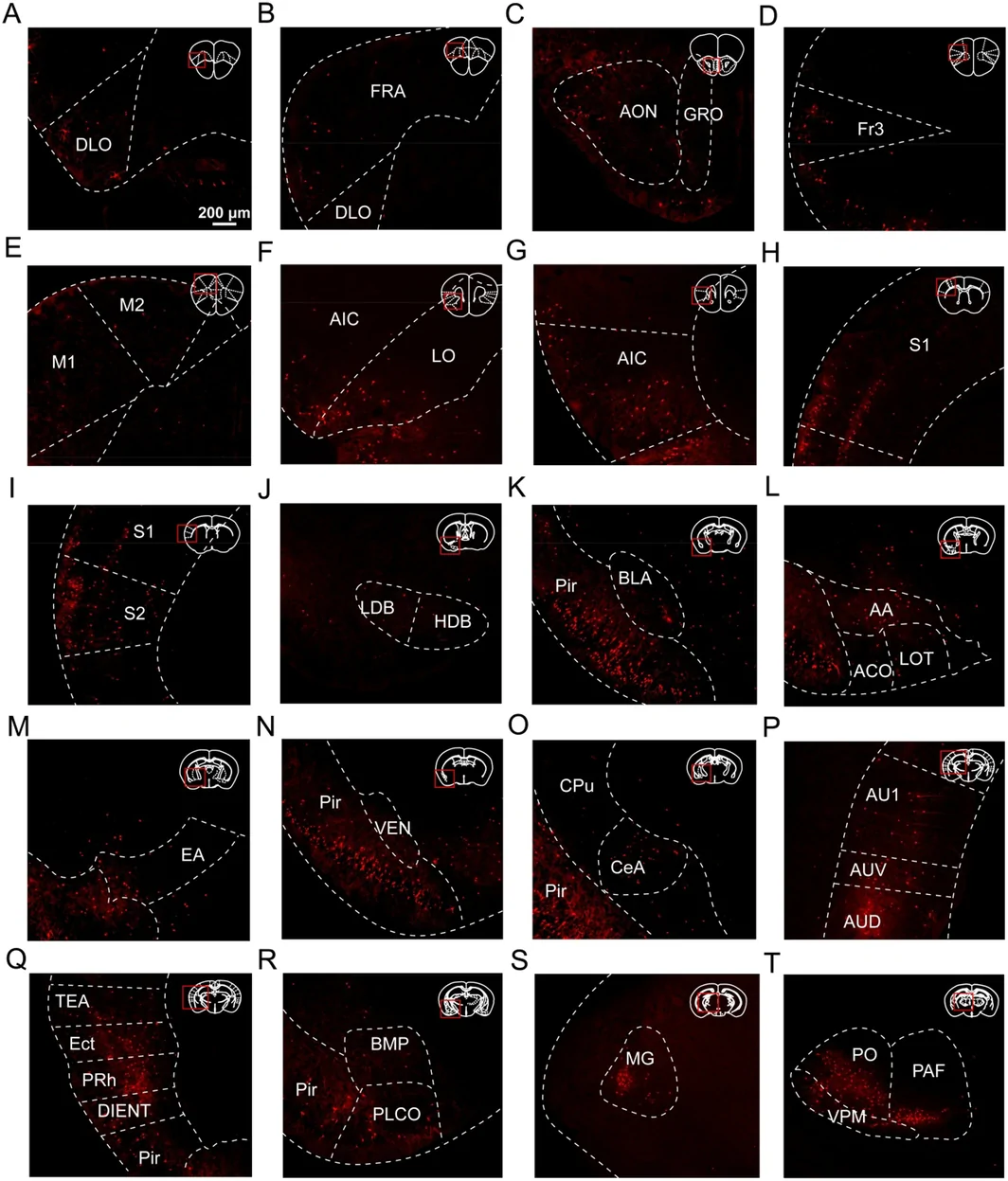
Representative nuclei with monosynaptic inputs to PIC GABAergic neurons. AA, anterior amygdaloid area; ACO, anterior cortical amygdaloid nucleus; AIC, anterior insular cortex; AON, anterior olfactory nucleus; AU1, primary auditory cortex; AUV, ventral secondary auditory cortex; BLA, basolateral amygdaloid nucleus; BMP, posterior basomedial amygdaloid nucleus; CeA, central amygdaloid nucleus; DIENT, dorsal intermediate entorhinal cortex; DLO, dorsolateral orbital cortex; EA, extension of the amygdala; Ect, ectorhinal cortex; Fr3, frontal cortex, area 3; FRA, frontal association cortex; GRO, granule cell layer of the olfactory bulb; HDB, horizontal limb of the diagonal band; LDB, lateral nucleus of the diagonal band; LO, lateral orbital cortex; LOT, nucleus of the lateral olfactory tract; M1, primary motor cortex; M2, secondary motor cortex; MG, medial geniculate nucleus; PAF, parafascicular thalamic nucleus; Pir, piriform cortex; PLCO, posterolateral cortical amygdaloid area; PO, posterior thalamic nuclear group; PRh, perirhinal cortex; S1, primary somatosensory cortex; S2, secondary somatosensory cortex; TEA, temporal association cortex; VEN, ventral entopiriform nucleus; VPM, ventral posteromedial thalamic nucleus.

DsRed‐labeled presynaptic inputs were predominantly in the telencephalon (the olfactory area, isocortex, basal ganglia, and amygdala) with additional inputs from the thalamus and midbrain, showing an ipsilateral preference. Some brain regions, such as S1, S2, primary motor cortex (M1), and secondary motor cortex (M2), exhibited bilateral labeling (Figure S3). Figure 2 shows magnified views of inputs from representative subregions, including: granule cell layer olfactory bulb (GRO) and nucleus of the lateral olfactory tract (LOT) in the olfactory areas; dorsolateral orbital cortex (DLO), frontal association cortex (FRA), anterior olfactory nucleus (AON), frontal cortex, area 3 (Fr3), primary motor cortex (M1), secondary motor cortex (M2), lateral orbital cortex (LO), anterior insular cortex (AIC), primary somatosensory cortex (S1), secondary somatosensory cortex (S2), ventral entopiriform nucleus (VEN), primary auditory cortex (AU1), ventral secondary auditory cortex (AUV), dorsal secondary auditory cortex (AUD), temporal association cortex (TEA), ectorhinal cortex (Ect), perirhinal cortex (PRh), dorsal intermediate entorhinal cortex (DIENT), posterolateral cortical amygdaloid area (PLCO), and piriform cortex (Pir) in the isocortex (Figure 2A–I,P,Q). The nucleus of the horizontal limb of the diagonal band (HDB), lateral nucleus of the diagonal band (LDB), and caudate putamen (CPu) in the basal ganglia provided inputs to PIC GABAergic neurons (Figure 2J,O). In the amygdala, PIC GABAergic neurons received inputs from the anterior amygdaloid area (AA), anterior cortical amygdaloid nucleus (ACO), extension of the amygdala (EA), central amygdaloid nucleus (CeA), basolateral amygdaloid nucleus (BLA), and posterior basomedial amygdaloid nucleus (BMP) (Figure 2K–M,O). In addition, the medial geniculate nucleus (MG), ventral posteromedial thalamic nucleus (VPM), posterior thalamic nuclear group (PO), and parafascicular thalamic nucleus (PAF) in the thalamus sent inputs to PIC GABAergic neurons (Figure 2S,T). Furthermore, there are also nuclei with fewer inputs, including dorsal endopiriform nucleus (DEN) and intermediate endopiriform nucleus (IEN) in the isocortex (Figure S4A); ventral pallidum (VP) and external globus pallidus (GPe) in the basal ganglia (Figure S4B,D). The interstitial nucleus of the posterior limb of the anterior commissure (IPAC), medial amygdaloid nucleus (MEA), anterior basomedial amygdaloid nucleus (BMA), and lateroanterior amygdaloid nucleus (LA) in the amygdala sent projections to PIC GABAergic neurons (Figure S4C,F,H). The anterior paraventricular thalamic nucleus (PVA) in the thalamus and the inferior colliculus (IC) in the midbrain provided inputs to PIC GABAergic neurons (Figure S4E,G). We have also calculated the convergence index (the ratio between the number of inputs and starters), which ranged between 35 and 45 (Figure S2B), a value related to the transynaptic efficiency of the virus [13]. The variability in the convergence index was similar to other rabies tracing studies [27].

### 3.3 Prevalence of Input Neurons Innervating PIC GABAergic Neurons

**FIGURE 3:**
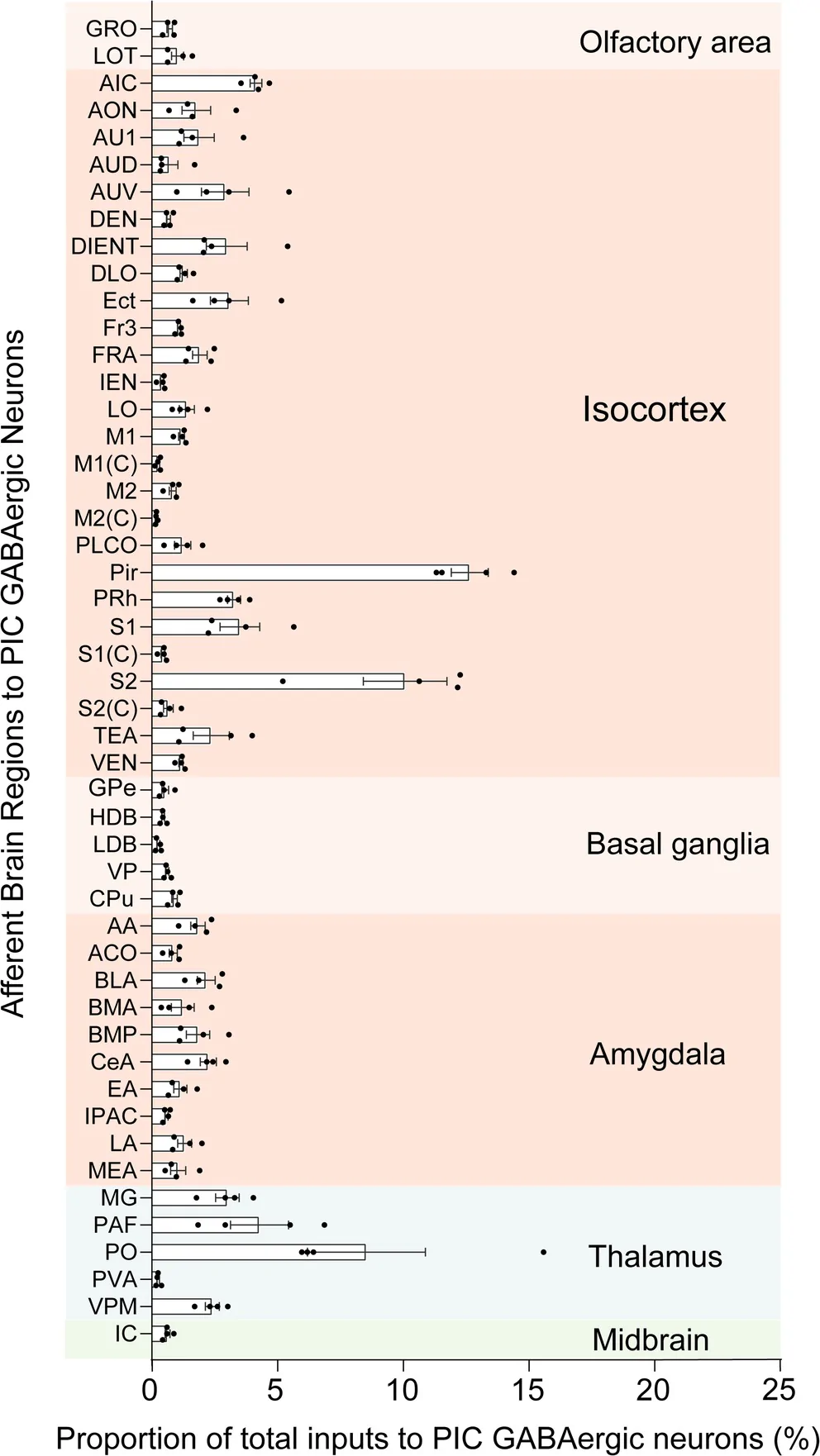
Prevalence of whole‐brain presynaptic inputs to PIC GABAergic neurons. The average proportion of dsRed‐labeled neurons was examined in all ipsilateral and contralateral brain regions providing more than 0.1% of the total afferent inputs to PIC GABAergic neurons (*n* = 4). These brain regions were classified into three main structures and four substructures. AA, anterior amygdaloid area; ACO, anterior cortical amygdaloid nucleus; AIC, anterior insular cortex; AON, anterior olfactory Nucleus; AU1, primary auditory cortex; AUD, dorsal secondary auditory cortex; AUV, ventral secondary auditory cortex; BLA, basolateral amygdaloid nucleus; BMA, anterior basomedial amygdaloid nucleus; BMP, posterior basomedial amygdaloid nucleus; CeA, central amygdaloid nucleus; CPu, caudate putamen; DEN, dorsal endopiriform nucleus; DIENT, dorsal intermediate entorhinal cortex; DLO, dorsolateral orbital cortex; EA, Extension of the amygdala; Ect, ectorhinal cortex; Fr3, frontal cortex, area 3; FRA, frontal association cortex; GPe, external globus pallidus; GRO, granule cell layer of the olfactory bulb; HDB, nucleus of the horizontal limb of the diagonal band; IC, inferior colliculus; IEN, intermediate endopiriform nucleus; IPAC, interstitial nucleus of the posterior limb of the anterior commissure; LA, lateroanterior hypothalamic nucleus; LDB, lateral nucleus of the diagonal band; LO, lateral orbital cortex; LOT, nucleus of the lateral olfactory tract; M1, primary motor cortex; M1(C), contralateral primary motor cortex; M2, secondary motor cortex; M2(C), contralateral secondary motor cortex; MEA, medial amygdaloid nucleus; MG, medial geniculate nucleus; PAF, parafascicular thalamic nucleus; Pir, piriform cortex; PLCO, posterolateral cortical amygdaloid area; PO, posterior thalamic nuclear group; PRh, perirhinal cortex; PVA, paraventricular thalamic nucleus, anterior part; S1, primary somatosensory cortex; S1(C), contralateral primary somatosensory cortex; S2, secondary somatosensory cortex; S2(C), contralateral secondary somatosensory cortex; TEA, temporal association cortex; VEN, ventral entopiriform nucleus; VP, ventral pallidum; VPM, ventral posteromedial thalamic nucleus. Data represent the mean ± SEM.

Brain regions with > 0.1% of total labeled neurons were defined as connected. A total of 45 nuclei were identified. We further analyzed the proportion of input neurons contributed by each brain region relative to the total labeled input neurons (Figure 3). The isocortex (62.08%) and amygdala (14.27%) provided the highest inputs. Within the isocortex, the densest inputs came from Pir (12.65% ± 0.74%), S2 (10.08% ± 1.67%), AIC (4.15% ± 0.23%), S1 (3.51% ± 0.79%), PRh (3.27% ± 0.26%), Ect (3.09% ± 0.75%), DIENT (2.98% ± 0.81%), AUV (2.93% ± 0.94%), and TEA (2.37% ± 0.72%). Contralateral inputs were also observed in the isocortex, including contralateral S1 (0.44% ± 0.08%), contralateral S2 (0.66% ± 0.19%), contralateral M1 (0.26% ± 0.05%), and contralateral M2 (0.19% ± 0.02%). Within the amygdala, CeA (2.25% ± 0.32%) and BLA (2.17% ± 0.35%) were the major input nuclei. Fewer inputs originated from the olfactory areas (GRO, 0.71% ± 0.11%; LOT, 1.03% ± 0.24%) and basal ganglia (VP, 0.62% ± 0.06%; CPu, 0.91% ± 0.11%).

In the thalamus, the top inputs were the PO (8.54% ± 0.35%), PAF (4.29% ± 1.16%), MG (3.01% ± 0.47%), and VPM (2.41% ± 0.28%).

In the midbrain, only sparse inputs were detected in the IC (0.62% ± 0.09%).

### 3.4 Downstream Output of PIC GABAergic Neurons

**FIGURE 4:**
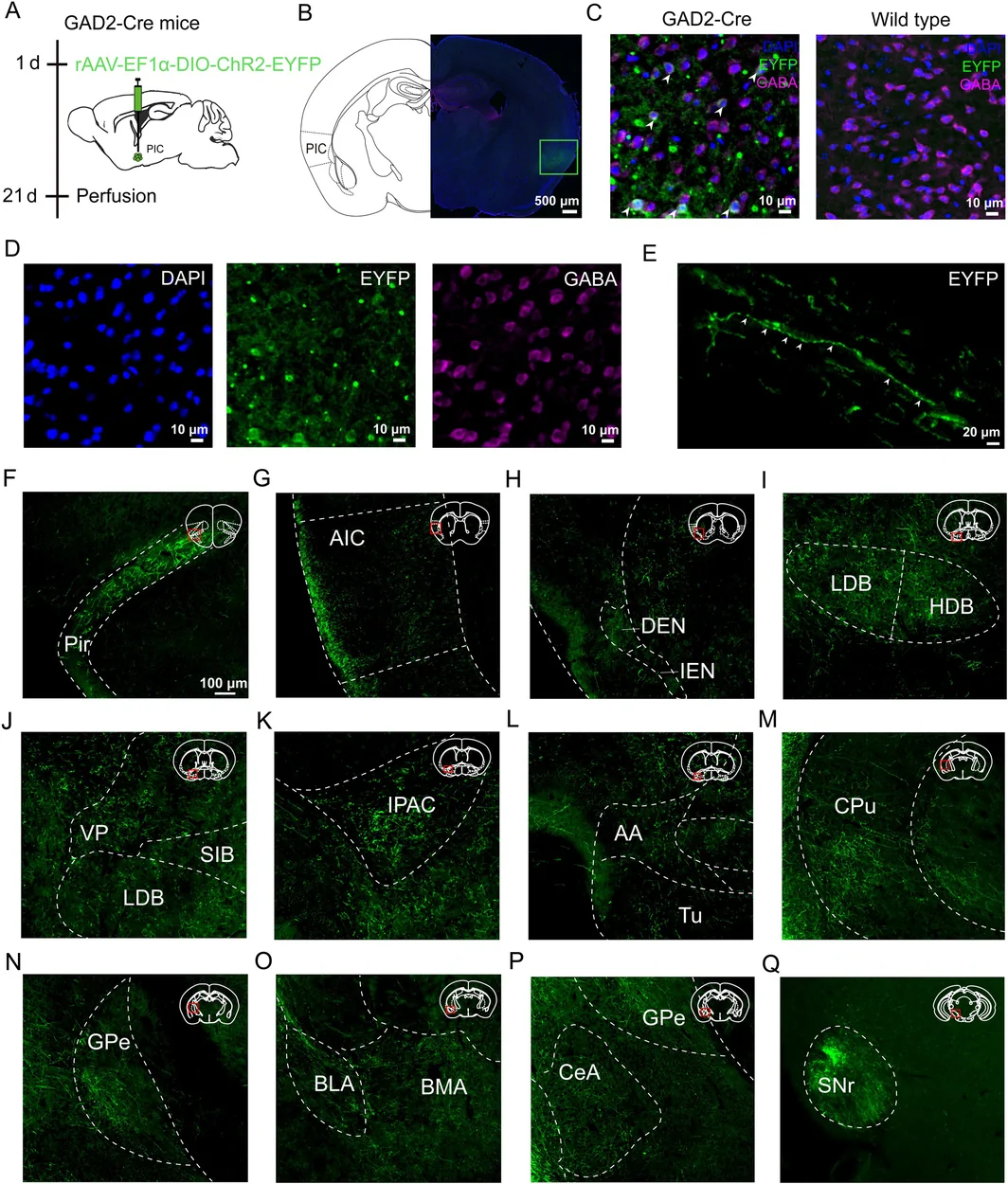
Viral tracing strategy and axonal projection patterns of PIC GABAergic neurons. (A) Schematic of the viral vector strategy. (B) Schematic localization of the viral injection site (green box) in GAD2‐IRES‐Cre mice brain. (C) Representative immunofluorescence images showing expression of DAPI (blue), eYFP (green), GABA (pink) and merge (arrow) in the PIC of GAD2‐IRES‐Cre mice (left) and WT mice (right). (D) High‐magnification view of the left panel in (C), showing individual channels for DAPI (blue), eYFP (green), and GABA (pink) signals. (E) Enlarged view of axonal puncta of GABAergic neurons (arrow). (F–Q) Exemplary targets of PIC GABAergic axonal projections. AA, anterior amygdaloid area; Ect, ectorhinal cortex; GPe, external globus pallidus; HDB, horizontal limb of the diagonal band; IPAC, interstitial nucleus of the posterior limb of the anterior commissure; LDB, lateral nucleus of the diagonal band; Pir, piriform cortex; PRh, perirhinal cortex; SIB, substantia innominata, basal; SNr, substantia nigra reticulata; Tu, olfactory tubercle; VP, ventral pallidum.

We next performed anterograde tracing to visualize the axonal projections of PIC GABAergic neurons to downstream brain regions using the Cre‐dependent rAAV virus expressing EYFP, thereby mapping the output targets of PIC GABAergic neurons [18, 19, 20]. To label axonal projections, rAAV‐EF1α‐DIO‐ChR2‐EYFP was injected into the PIC region of GAD2‐IRES‐Cre mice for anterograde tracing. Twenty‐one days after viral injection, the mice were transcardially perfused and brains were collected (Figure 4A). EYFP‐labeled neurons were observed in the PIC (Figure 4B). Immunofluorescence staining of brain sections from GAD2‐IRES‐Cre mice with an anti‐GABA antibody revealed colocalization of eYFP and GABA signals. In contrast, no fluorescent labeling was detected in brain sections from WT mice, confirming the specificity of the viral labeling strategy for GABAergic neurons (Figure 4C,D). Only axons with varicosities were considered as representing downstream projection regions; straight fibers lacking such varicosities were excluded, as they were likely fibers of passage (Figure 4E) [25].

### 3.5 Output Patterns of GABAergic Neurons in the PIC

The efferent projections were distributed in an ipsilateral manner, and the results showed that axonal projections were primarily located in a total of 18 nuclei across the telencephalon and midbrain. In the telencephalon, projections targeted the olfactory area (olfactory tubercle, Tu), isocortex, basal ganglia, and amygdala (Figure 4F–Q). Axonal projections in the isocortex primarily targeted the AIC, IEN, DEN, and Pir (Figure 4F–H). In the basal ganglia, the HDB, LDB, VP, Substantia innominata basal (SIB), CPu and GPe received projections (Figure 4I,J,M,N). Additionally, PIC GABAergic neurons sent dense projections to the IPAC, AA, CeA, LA, BLA, and BMA in the amygdala (Figure 4K,L,O,P). In the midbrain, only the substantia nigra reticulata (SNr) received projections (Figure 4Q).

### 3.6 Prevalence of Whole‐Brain Outputs of PIC GABAergic Neurons

**FIGURE 5:**
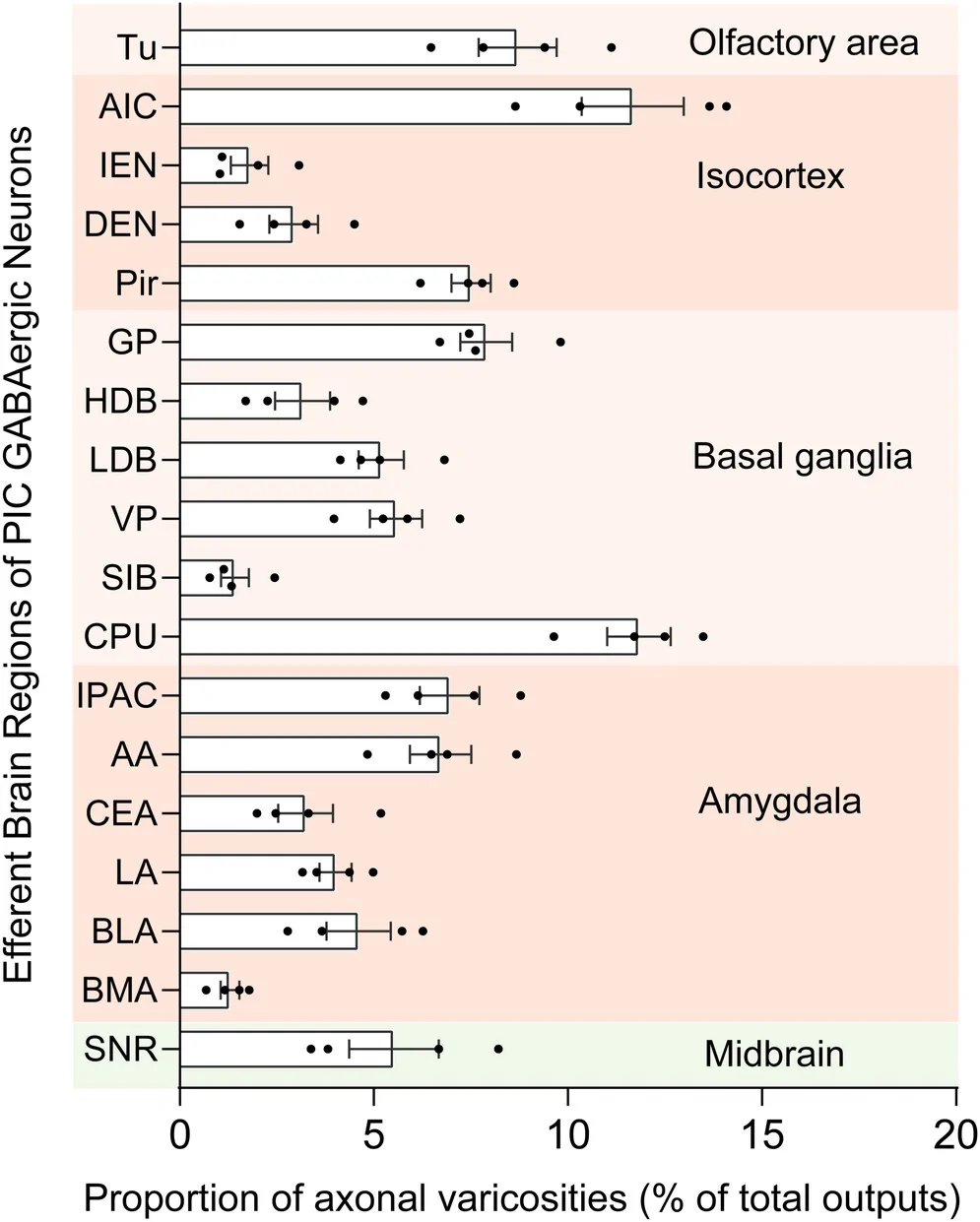
Prevalence of whole‐brain outputs of PIC GABAergic neurons. For each target region, the proportion of axonal varicosities was calculated relative to the total number of whole‐brain outputs. These brain regions were classified into two main structures and four substructures. AA, anterior amygdaloid area; GPe, external globus pallidus; HDB, horizontal limb of the diagonal band; IPAC, interstitial nucleus of the posterior limb of the anterior commissure; LDB, lateral nucleus of the diagonal band; Pir, piriform cortex; PRh, perirhinal cortex; SIB, substantia innominata, basal; SNr, substantia nigra reticulata; Tu, olfactory tubercle; VP, ventral pallidum. Data represent the mean ± SEM.

The proportion of axonal varicosities in each nucleus was calculated as the ratio of the number of EYFP‐labeled varicosities in each nucleus to the total number of EYFP‐labeled varicosities [21, 24]. Brain regions with > 0.1% of total labeled varicosities were defined as connected. The results revealed that efferent projections from PIC GABAergic neurons predominantly targeted the telencephalon and midbrain (Figure 5). Within the telencephalon, projections to the basal ganglia accounted for approximately 35.06% of the total, with densest innervation to CPu (11.83% ± 0.82%), GPe (7.90% ± 0.67%), VP (5.57% ± 0.68%), and LDB (5.20% ± 0.58%). Projections to the amygdala and isocortex constituted 26.67% and 25.31%, respectively. Within the amygdala, the IPAC (6.95% ± 0.77%), AA (6.72% ± 0.79%), BLA (4.61% ± 0.83%), LA (4.01% ± 0.41%), and CeA (3.24% ± 0.70%) received major inputs. In the isocortex, major projections were observed in AIC (11.67% ± 1.31%) and Pir (7.50% ± 0.50%). Projections were also found to innervate the olfactory areas (Tu, 8.70% ± 1.00%) and midbrain (SNr, 5.52% ± 1.16%).

### 3.7 Comparison of Inputs and Outputs of PIC GABAergic Neurons

**FIGURE 6:**
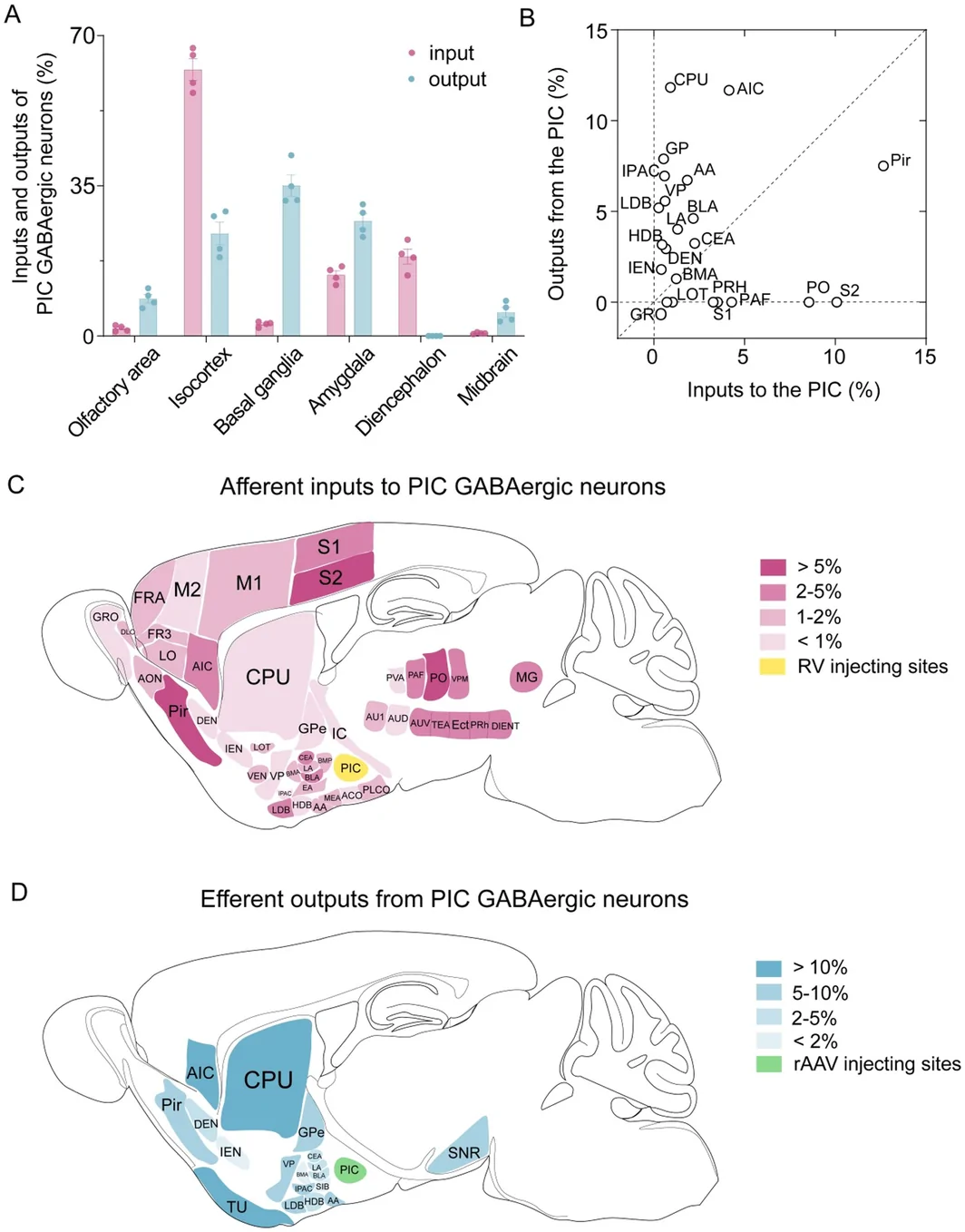
Comparative analysis of inputs and outputs of PIC GABAergic neurons. (A) Comparative analysis of the proportions of inputs (red) and outputs (blue) of PIC GABAergic neurons in the six main structures of the whole brain. Values are means ± SEM (*n* = 4). (B) The proportion of input versus output in each region of the typical nuclei associated with the PIC. (C, D) Sagittal schematic diagrams depicting (C) the afferent inputs to and (D) the efferent outputs from GABAergic neurons within the PIC. The color‐coded transparency indicates the percentage distribution of these inputs and outputs. AA, anterior amygdaloid area; ACO, anterior cortical amygdaloid nucleus; AIC, anterior insular cortex; AON, accessory olfactory tract; AU1, primary auditory cortex; AUD, dorsal secondary auditory cortex; AUV, ventral secondary auditory cortex; BLA, basolateral amygdaloid nucleus; BMA, anterior basomedial amygdaloid nucleus; BMP, posterior basomedial amygdaloid nucleus; CeA, central amygdaloid nucleus; CPu, caudate putamen; DEN, dorsal endopiriform nucleus; DIENT, dorsal intermediate entorhinal cortex; DLO, dorsolateral orbital cortex; EA, extension of the amygdala; Ect, ectorhinal cortex; Fr3, frontal cortex, area 3; FRA, frontal association cortex; GPe, external globus pallidus; GRO, granule cell layer of the olfactory bulb; HDB, nucleus of the horizontal limb of the diagonal band; IC, inferior colliculus; IEN, intermediate endopiriform nucleus; IPAC, interstitial nucleus of the posterior limb of the anterior commissure; LA, lateroanterior hypothalamic nucleus; LDB, lateral nucleus of the diagonal band; LO, lateral orbital cortex; LOT, nucleus of the lateral olfactory tract; M1, primary motor cortex; M2, secondary motor cortex; MEA, medial amygdaloid nucleus; MG, medial geniculate nucleus; PAF, parafascicular thalamic nucleus; Pir, piriform cortex; PLCO, posterolateral cortical amygdaloid area; PO, posterior thalamic nuclear group; PRh, perirhinal cortex; PVA, paraventricular thalamic nucleus, anterior part; S1, primary somatosensory cortex; S2, secondary somatosensory cortex; SNr, substantia nigra reticulata; SIB, substantia innominata, basal; TEA, temporal association cortex; Tu, olfactory tubercle; VEN, ventral entopiriform nucleus; VP, ventral pallidum; VPM, ventral posteromedial thalamic nucleus.

By comparing the afferents and efferents of PIC GABAergic neurons across brain subregions, we found that the isocortex and amygdala showed significant bidirectional connectivity with PIC GABAergic neurons, but with differential connectivity patterns: isocortical inputs (62.08%) considerably exceeded outputs (23.90%), while amygdala outputs (26.81%) exceeded inputs (14.27%) (Figure 6A). Inputs from olfactory areas, basal ganglia, and midbrain were considerably lower than outputs to these regions (olfactory: 1.74% vs. 8.70%; basal ganglia: 2.77% vs. 35.06%; midbrain: 0.63% vs. 5.52%). Notably, the thalamus provided dense inputs but received no outputs. Collectively, these comparative analyses revealed that although PIC GABAergic neurons receive the highest proportion of inputs from the isocortex and thalamus, their densest axonal projections target the basal ganglia, followed by the amygdala. Notably, PIC GABAergic neurons received substantial inputs from the thalamus but did not send any axonal projections to this structure.

Next, we performed a comparative analysis of input and output proportions for individual nuclei. The results showed that PIC GABAergic neurons exhibited similar reciprocal connectivity proportions with the BMA (Figure 6B; input, 1.23% ± 0.45%; output, 1.29% ± 0.24%). The Pir received prominent inputs (12.65%) and also sent substantial outputs (7.50%). The AIC received moderate inputs (4.15%) but sent dense outputs (11.67%). Other input‐dominant nuclei (S1/S2, PO, PAF) received minimal outputs. Output‐dominant nuclei (CPu, GPe, VP, IPAC, AA, BLA) sent weaker inputs back. Figure 6C,D present a systematic summary of the afferents and efferents of PIC GABAergic neurons. The schematic diagram of the neuronal distribution clearly illustrates the anatomical characteristics of this innervation.

## 4 Discussion

PIC GABAergic neurons are involved in pain [28, 29], memory [7], and stress [6, 30]. Understanding their circuits requires mapping connectivity. While the existence of long‐range GABAergic projection neurons is now established across diverse cortical areas [31, 32, 33, 34], their presence and functional role in the posterior insular cortex remain, to our knowledge, undescribed. We mapped whole‐brain inputs and outputs. They bidirectionally connect with isocortex (input‐dominant), receive unidirectional thalamic inputs, and send high‐contribution projections to basal ganglia and amygdala (output‐dominant). At the nucleus level, Pir, S1/S2, PO, PAF show input predominance, while major outputs target CPu, GPe, VP, IPAC, AA, BLA. These patterns suggest PIC GABAergic neurons integrate sensory/thalamic information and transmit to downstream regions involved in sensory, sleep, emotion, and motor regulation, acting as a multimodal hub, though this needs further testing. This provides anatomical evidence for their regulatory roles.

### 4.1 Methodological Strengths of Trans‐Synaptic Tracing Over Traditional Retrograde Approaches

Using cell‐type‐specific RV tracing, we identified 45 input nuclei (including novel olfactory/midbrain inputs) and sparse contralateral cortical projections. AAV tracing revealed 18 downstream nuclei. At the same time, we have noticed that the discrepancy with Gehrlach et al. [1], who reported no long‐range projections. This likely reflects methodological differences: we used AAV2/9 (broader diffusion/higher transport) versus AAV2/5 [35, 36], EF1α‐promoter (less silencing) [37] driving ChR2‐EYFP (membrane‐targeted) [18, 19, 20], and low titer excludes load effects. Thus pIC projections are genuine but sparse, requiring sensitive detection. Our approach offers high‐resolution mapping.

### 4.2 Involvement of PIC GABAergic Neurons in Sensory Processing

PIC GABAergic neurons play a key role in sensory information processing, including pain and thermal perception. In recent years, multiple lines of clinical evidence have implicated PIC GABAergic neurons in pain perception [28, 38, 39]. In this study, we found that PIC GABAergic neurons receive dense input from the Pir [40], S1/S2 [41], AIC [42], and LO [43] which regulate pain perception. Notably, the bidirectional connectivity with Pir and AIC observed in our study raises the hypothetical possibility of a negative feedback loop maintaining excitation/inhibition balance, although this remains untested. Meanwhile, the CeA, which has connections with the PIC, also regulates pain‐related behaviors [44, 45].

For thermal perception, the spinothalamocortical pathway via the thalamus to S1 and PIC [46, 47] is consistent with our tracing. This suggests involvement in temperature encoding, but direct functional evidence is lacking.

Furthermore, PIC GABAergic neurons are also connected to multiple sensory cortices, including the Pir, Tu, and the TEA [48, 49]. These connections provide a neuroanatomical foundation for hypothesizing their potential involvement in multimodal sensory integration, such as vision, audition, and olfaction. However, whether they directly regulate these processes requires further investigation. Overall, these neural circuits offer testable hypotheses for future studies exploring the role of PIC GABAergic neurons in broader sensory information processing.

### 4.3 Potential Involvement of the PIC GABAergic in Sleep–Wake Regulation Based on Neuroanatomical Evidence

Recent fMRI studies link poor sleep quality to decreased insular perfusion [50] and increased insular functional connectivity with short sleep duration and depressive symptoms [51, 52], but the direct role of PIC GABAergic remains unknown.

Our findings show PIC GABAergic neurons connect with multiple brain regions involved in sleep–wake regulation, including the amygdala (CeA, BLA) and the basal ganglia (CPu, GPe, VP). Based on the known functions of these regions [53, 54, 55, 56, 57, 58, 59], these anatomical connections raise the possibility that PIC GABAergic neurons could participate in sleep–wake regulation, though direct evidence is absent. In addition to the potential direct modulation of sleep by PIC GABAergic neurons, sensory inputs such as pain and temperature changes may also influence sleep–wake behavior, as exemplified by the inputs received from the TEA region, which is associated with sound‐mediated arousal [60]. Future studies may test whether PIC GABAergic neurons mediate these interactions and whether this involves integration of thalamic or cortical signals. The specific regulatory mechanisms and underlying neural pathways remain to be further elucidated.

### 4.4 The Functional Implications of PIC GABAergic Neurons in Emotion‐Related Processes

PIC GABAergic neurons are critically involved in various emotional regulation processes, encompassing stress responses [6, 61], fear memories consolidation, and affective states such as anxiety and depression. In this study, we found that PIC GABAergic neurons receive inputs from stress‐regulating regions such as thalamic PO, PAF, and MG (audiogenic stress) [62, 63, 64], suggesting these circuits may participate in the modulation of audiogenic stress behaviors. Additionally, the CeA [65, 66], BLA [67], and EA [67] in the amygdala, which are involved in stress regulation, exhibit bidirectional connectivity with PIC GABAergic neurons. The CeA‐PIC glutamatergic circuit mediates aversive emotions [68]. Inputs from isocortical FRA, AU1, and AUD in isocortex [69, 70, 71] also regulate stress.

PIC GABAergic neurons are a necessary part of the neural circuitry related to the consolidation of cued‐fear memories [7, 72]. In our study, bidirectionality with BLA [73], LA [74], VP [75] supports involvement. Many connected nuclei regulate anxiety/depression, including the PO [76] and PAF [63] in the thalamus, the AIC [77] and AUD [71] in the isocortex, the CeA [78], BLA [79], and LA [80] in the amygdala, and the VP [59] in the basal ganglia. These anatomical connections suggest PIC GABAergic neurons may form circuits with these nuclei to regulate emotion, though direct evidence for anxiety/depression is lacking.

### 4.5 Functional Considerations of PIC GABAergic Neurons in Motor Regulation

PIC GABAergic neurons connect with motor‐related nuclei: thalamic PO and PAF [81, 82], isocortical S1/S2 and M1/M2 [83, 84, 85], and basal ganglia CPu and GPe [86], suggesting a potential motor role that remains unexplored. Future studies combining circuit tracing, calcium imaging, and behavioral assays are needed.

### 4.6 Limitations

Several limitations related to our tracing tools and data interpretation should be explicitly considered. One limitation of our retrograde tracing is the RV tracing has cytotoxicity; despite a standard one‐week survival, sublethal toxicity may underestimate inputs. Future studies using complementary tracers (e.g., CTB, AAV, or nontoxic viral tools) could help validate our map. Nevertheless, the strong consistency with previous reports [1] supports the reliability of our data.

A potential concern in AAV‐based anterograde tracing is retrograde transduction. In all animals analyzed, we found no EYFP‐positive somata outside the PIC injection site, consistent with AAV2/9 properties [35] and Cre‐dependence. ChR2‐EYFP membrane‐targeting may overrepresent varicosities relative to synapses, so we interpret them as a relative projection density index, not an absolute synapse count; uniform parameters ensure valid comparisons. Finally, functional roles remain speculative and require experimental validation.

## 5 Conclusion

This study revealed that PIC GABAergic neurons receive dense inputs from the isocortex and thalamic nuclei, and send dominant projections downstream to the basal ganglia and amygdala, with these brain regions constituting the core hub of their whole‐brain connectivity. PIC GABAergic neurons play important roles in various physiological functions, including sleep–wake regulation, thermal and pain perception, and emotional regulation. The neural circuits identified here provide an anatomical basis for understanding these functions and for treating neurological disorders associated with PIC dysfunction.

## Author Contributions

W.‐Q.Q. and X.‐Y.Z.: experimental operations, data collection and analysis, figure preparation, manuscript drafting/revising. J.‐Q.S.: data analysis. W.‐X.M. and W.‐M.Q.: experiment execution. Z.‐L.H. and Y.‐L.Z.: study concept/design, manuscript drafting/revising. All authors read and approved the final version.

## Funding

The authors have nothing to report.

## Disclosure

The authors have nothing to report.

## Ethics Statement

All procedures were approved by the Animal Ethics Committee of the Second Affiliated Hospital of Harbin Medical University (reference number: YJSDW2024‐118).

## Conflicts of Interest

The authors declare no conflicts of interest.

## Supporting information


**Figure S1:** Mapping of the starter neurons in the PIC. Coronal sections showing the distribution of starter neurons in the PIC (between +0.37 and −1.31 mm from bregma) of four GAD2‐IRES‐Cre mice. The images were reconstructed in ImageJ to identify brain regions based on the reference brain atlas; starter cells are indicated by the orange points and the shaded region.
**Figure S2:** The relationship between the numbers of starter cells and input cells. (A) The total number of starter cells for each brain sample. (B) The convergence index (input cell count/starter cell count) for each brain sample.
**Figure S3:** Representative nuclei with contralateral monosynaptic inputs to PIC GABAergic neurons. M1(C), Contralateral primary motor cortex; M2(C), Contralateral secondary motor cortex; S1(C), Contralateral primary somatosensory cortex; S2(C), Contralateral secondary somatosensory cortex.
**Figure S4:** Representative nuclei with sparse monosynaptic inputs to PIC GABAergic neurons. AUD, dorsal secondary auditory cortex; BMA, anterior basomedial amygdaloid nucleus; CPU, caudate putamen; DEN, dorsal endopiriform nucleus; GPe, external globus pallidus; IC, inferior colliculus; IEN, intermediate endopiriform nucleus; IPAC, interstitial nucleus of the posterior limb of the anterior commissure; LA, lateroanterior hypothalamic nucleus; MEA, medial amygdaloid nucleus; PVA, anterior paraventricular thalamic nucleus; VP, ventral pallidum.

## Data Availability

All data supporting the conclusions are included in the main text and Supporting Information. Additional datasets are available from the corresponding author upon reasonable request.
