# Supplementary material for: Whole‐Brain Monosynaptic Inputs to and Axonal Projections From Posterior Insular Cortex GABAergic Neurons in Mice
Source: CNS Neurosci Ther. 2026 Sep 4;32(9):e71102. doi: 10.1002/cns.71102 (PMC13545509; doi:10.1002/cns.71102)
Supplement: Supplementary file 1 [file CNS-32-e71102-s001.docx]

Figure S1. Mapping of the starter neurons in the PIC. Coronal sections showing the distribution of starter neurons in the PIC (between +0.37 mm and -1.31 mm from bregma) of four GAD2-IRES-Cre mice. The images were reconstructed in ImageJ to identify brain regions based on the reference brain atlas; starter cells are indicated by the orange points and the shaded region

Figure S2. The relationship between the numbers of starter cells and input cells. (A) The total number of starter cells for each brain sample. (B) The convergence index (input cell count/starter cell count) for each brain sample.

Figure S3. Representative nuclei with contralateral monosynaptic inputs to PIC GABAergic neurons. Abbreviations: M1(C), Contralateral primary motor cortex; M2(C), Contralateral secondary motor cortex; S1(C), Contralateral primary somatosensory cortex; S2(C), Contralateral secondary somatosensory cortex.

Figure S4. Representative nuclei with sparse monosynaptic inputs to PIC GABAergic neurons. Abbreviations: AUD, Dorsal secondary auditory cortex; BMA, Anterior basomedial amygdaloid nucleus; CPU, Caudate putamen; DEN, Dorsal endopiriform nucleus; GPe, External globus pallidus; IC, Inferior colliculus; IEN, Intermediate endopiriform nucleus; IPAC, Interstitial nucleus of the posterior limb of the anterior Commissure; LA, Lateroanterior hypothalamic nucleus; MEA, Medial amygdaloid nucleus; PVA, Anterior paraventricular thalamic nucleus; VP, Ventral pallidum.
